# Individualized, discrete event, simulations provide insight into inter- and intra-subject variability of extended-release, drug products

**DOI:** 10.1186/1742-4682-9-39

**Published:** 2012-08-31

**Authors:** Sean HJ Kim, Andre J Jackson, Rim Hur, C Anthony Hunt

**Affiliations:** 1 Department of Bioengineering and Therapeutic Sciences, University of California, San Francisco, CA, USA; 2 Office of Clinical Pharmacology, Food and Drug Administration, Washington, DC, USA

## Abstract

**Objective:**

Develop and validate particular, concrete, and abstract yet plausible in silico mechanistic explanations for large intra- and interindividual variability observed for eleven bioequivalence study participants. Do so in the face of considerable uncertainty about mechanisms.

**Methods:**

We constructed an object-oriented, discrete event model called subject (we use small caps to distinguish computational objects from their biological counterparts). It maps abstractly to a dissolution test system and study subject to whom product was administered orally. A subject comprises four interconnected grid spaces and event mechanisms that map to different physiological features and processes. Drugs move within and between spaces. We followed an established, Iterative Refinement Protocol. Individualized mechanisms were made sufficiently complicated to achieve prespecified Similarity Criteria, but no more so. Within subjects, the dissolution space is linked to both a product-subject Interaction Space and the GI tract. The GI tract and Interaction Space connect to plasma, from which drug is eliminated.

**Results:**

We discovered parameterizations that enabled the eleven subject simulation results to achieve the most stringent Similarity Criteria. Simulated profiles closely resembled those with normal, odd, and double peaks. We observed important subject-by-formulation interactions within subjects.

**Conclusion:**

We hypothesize that there were interactions within bioequivalence study participants corresponding to the subject-by-formulation interactions within subjects. Further progress requires methods to transition currently abstract subject mechanisms iteratively and parsimoniously to be more physiologically realistic. As that objective is achieved, the approach presented is expected to become beneficial to drug development (e.g., controlled release) and to a reduction in the number of subjects needed per study plus faster regulatory review.

## Background

Large intrasubject variability in drug bioequivalence (BE) coupled with weak in vitro-to-in vivo correlation can pose significant problems in assessing bioequivalence [1-3]. We observed examples of large intra- and interindividual variability in data from a bioequivalence study. A proposed strategy for exploring plausible explanations, one that has since been abandoned, was individual BE. The focus was to investigate important subject-by-formulation interactions, if they exist [4,5]. When faced with such data, an obvious question is, what are plausible, mechanistic, root causes of that variability? We used an unconventional modeling and simulation strategy to develop particular, concrete, parsimonious yet plausible abstract answers that strove to avoid accumulating tenuous assumptions. We present individualized answers in the form of subject-by-formulation interactions that emerged within models for a set of eleven subjects drawn from one BE study.

Plausible, conceptual explanations for such variability have been discussed [6]. Several pharmacokinetic modeling and simulation strategies have been offered for deciphering atypical drug absorption profiles, including using a sum of inverse Gaussian functions to describe absorption [7] as part of a parametric, nonlinear mixed effects analysis [8]. Such analyses may fail because mechanisms underlying the data contradict one or more of the assumptions on which the formal approach rests. Sparse data aggravates the problem. That problem can be solved when population mathematical descriptions within nonlinear mixed effect pharmacokinetic models can be expanded to cover more mechanistic assumptions [9-11]. When data are rich, the problem may be addressable using two-stage techniques [9], which allow more flexibility in specifying absorption characteristics of the structural model. However, if the failure is because different mechanisms (i.e., different structural models) seem to apply to subsets of individuals, but not on all occasions, then multiple assumptions made by such mathematical models are violated. In that case, even with rich data, such analyses cannot be relied upon to provide trustable mechanistic insight. The latter situation occurs for many complex controlled release formulations. Hénin et. al. [11] describe an example involving a complex felodipine tablet formulation, and discusses the problem from the conventional modeling perspective. In such situations, different methods, like those presented herein, are needed.

In concluding a review of methods of deciphering atypical drug absorption profiles, Zhou [6] opined, “it can be envisioned that … absorption analysis may move toward more mechanism-based rather than simply abstract number crunching. It may also be expected that more and more novel research techniques and computational tools will be used to greatly facilitate the in-depth understanding of absorption processes.” Such progress would expand the “personalized medicine” vision to include complicated oral dosage forms [10,12]. Before we can develop methods that provide exploitable explanations of atypical drug absorption profiles, we need means to begin achieving deeper, concrete insight into mechanisms that may underlie intra- and interindividual differences in bioavailability data, including subject-by-formulation interactions [5], when they exist.

**Figure 1:**
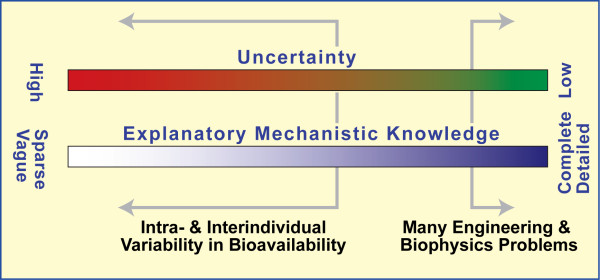
**Scales characterizing bioavailability studies.** Any feature or property of a specific study subject following dosing (the system) can be characterized by an approximate location on both scales. Uncertainty example: we know very little about the likely state of the extended release dosage in a particular subject at a particular time after dosing. Consequently, for that feature, we are left of center on the Uncertainty Scale. Mechanistic knowledge: we know very little about the actual mechanisms responsible for differences in drug plasma profiles. Consequently, here too, we are on the left side of the scale. We need plausible mechanisms that can explain the large intra- and interindividual variability in bioavailability.

Why do we need a modeling and simulation approach that is fundamentally different from conventional physiologically based and population pharmacokinetic approaches? The circumstances of a BE study can be characterized by indicating an approximate location on the two scales in Figure 1. For an established dosage form, for which we have repeated, good correlations between in vitro measures of dissolution and bioavailability measures, little intra-individual variability, and explainable interindividual variability, we would select locations somewhat right of center. Being on the far right (characteristic of many engineering problems) favors developing inductive models that can be precise, accurate, and predictive: the generators of underlying phenomena are well understood, ample quantitative data is available, and precise knowledge about mechanisms is available at all levels of granularity. One’s location shifts left when dealing with living systems because uncertainty increases and precise knowledge diminishes. Conceptual mechanisms are less validated (thus less trustworthy) and more hypothetical. The reliable, quantitative data that would be needed to validate (or falsify) even a modestly complicated, explanatory, mechanistic model are often lacking or scarce. When intra- and interindividual variability increases (e.g., complex, extended release formulations), one’s location shifts further left, and the risks and associated problems of relying on induction and inductive models begin accumulating. Yet the need for more complicated, particular (rather than generalized) individualized explanations increases. Prior to the introduction of object-oriented methods, there was no sound option but to continue relying on equation based, inductive models such as those used to study oral absorption [6,13,14]. The method for doing so is straightforward and effective under many circumstances, but hinges on an idealized scenario that enables moving far right in Figure 1, a scenario that is easily described by an equation-based model when some set of assumptions are met. Herein, we are not interested in idealized scenarios, so we elected to explore the approach described below.

We began answering the question posed above by discovering plausible, abstract yet concrete mechanistic explanations for eleven examples (exhibiting the most intra- and interindividual variability from a study involving 32 subjects) of intra-individual differences in bioavailability and its role in the determination of BE of a generic and originator product. For reasons stated above, we sought new methods that would provide the flexibility needed given considerable uncertainty.

We used object-oriented, discrete event, modeling and simulation methods to build concrete software devices composed of three or more discretized spaces and mobile objects (mapping to drug) that, when measured during simulation, mimic measured features of drug release from a dosage form along with important features of the plasma drug concentration versus time profiles. The device is an analogue of a subject participating in a bioequivalence study. Hunt et al. [15] describe how the approach is fundamentally different from conventional physiologically based and population pharmacokinetic approaches. Our objective was to discover separate, individualized analogues that produce “drug dissolution” and individual “disposition” profiles that closely match their counterparts, as determined by prespecified Similarity Criteria (SC). We adhered to a strong guideline: make each analogue and its mechanisms no more complicated than needed to achieve the SC. We conjectured that once targeted SC have been achieved for a given subject, we could hypothesize that in silico mechanistic details might have had BE-study-subject counterparts [15] at a comparable abstraction level. When simulation results fail to achieve the SC, we can state that the analogue and its mechanisms do not have real world counterparts.

Starting with a simple, prototypal analogue, we used an Iterative Refinement Protocol (IR Protocol) to improve similarity between in silico and corresponding subject plasma profiles. We used medium and stringent, multi-attribute SC. We evaluated three structurally different versions of subject, one simple and two somewhat more complicated. All three achieved the medium SC for all subjects. The gastro-intestinal (GI) component of each subject mapped to a non-homogeneous GI tract. The third subject, the focus of this report, had a two-component, heterogeneous, individualizable “GI tract.” Parameterizations were discovered that achieved the stringent SC for all eleven plasma profile pairs. Originator and test product mean dissolution profiles were different; a corresponding difference was built into subjects. To achieve the stringent SC, it was necessary to specify additional, modest intra- and interindividual differences in analogue counterparts to product dissolution. It was also necessary to specify both intra- and interindividual differences in drug disposition within subjects.

The parameterized subjects are simple and intuitive. Coarse-grained dynamic details can be observed during simulations. We hypothesize that all had BE study counterparts. In achieving the stringent SC, subject parameterizations and executions brought into clear focus plausible subject-by-formulation interactions. If evidence becomes available that falsifies one or more events or processes, it is straightforward to use the IR Protocol to make adjustments that reestablish validation. It is easy to conceptualize mappings from events occurring during simulations and counterparts occurring during product dissolution, drug absorption, and disposition within individual subjects. In that way, the simulations facilitate thinking more deeply about the real system. Insights gained from this new class of simulation models may lead to ideas for improving complicated formulations to achieve bioequivalence or enable controlled individualization of product performance.

## Methods

### Bioequivalence studies

A randomized, single-dose, two-way crossover study design under fasting conditions was used to evaluate the bioequivalence of drug X in originator and test, controlled release formulations. All 32 volunteers were healthy adults. Two subjects failed to complete the study resulting in a final N = 30, from which we selected eleven that exhibited especially large or atypical variability. A validated assay (liquid chromatography - mass spectrometry and liquid chromatography - tandem mass spectrometry) was used to determine drug X and its metabolite levels in plasma. The assay was linear between 1 and 400 ng/ml. The overall inter-day precision (% coefficient of variation) and accuracy for the standards and quality control samples were within the range of 2.4 to 9.3% and 92.4 to 108%, respectively. Dissolution studies were conducted using USP basket at 100 rpm in 900 ml of pH 7.2 buffer solutions at 37°C. Samples were taken at time 0, 0.5, 1, 1.5, 2, 4 hours and then every 2 hours thereafter to 24 hrs.

### In silico approach

**Figure 2:**
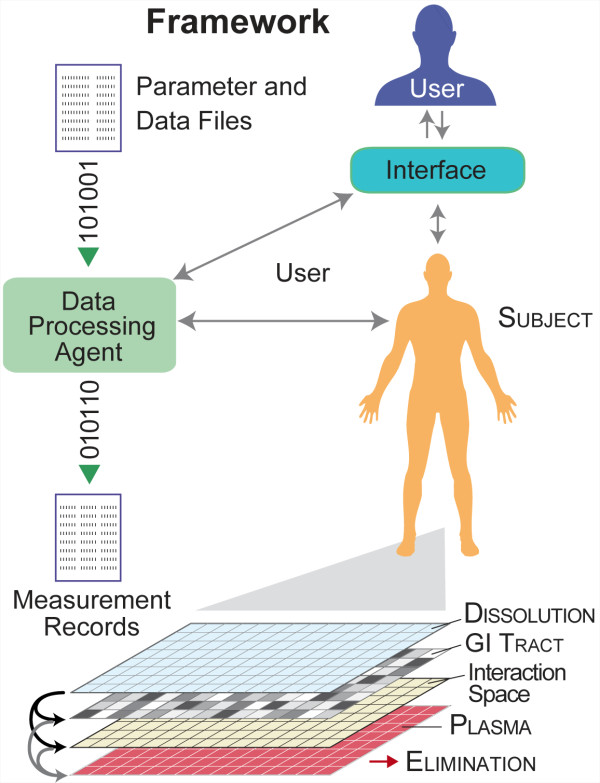
**Framework.** The system comprises a core, in silico model supported by framework features for simulation and analysis. The framework simulates whole-body drug disposition experiments. The basic design has three grid spaces that abstractly map to the dissolution compartment, GI tract, and plasma. The arrows indicate grid-to-grid connections. Drug objects move between the interconnected spaces, and exit from the plasma grid. Simulated diffusion occurs within each grid. GI tract can be represented using multiple grids to introduce structural and functional heterogeneity. A reservoir space is an option, which can be added and connected to GI tract. Spaces shaded differently within GI tract indicate that their properties can be customized, should that be needed. Supporting framework components include a Data Processing Agent and graphical user interface. The Data Processing Agent parses parameter files and referent data for simulation setup, and accesses subject during simulation to automatically record and analyze measurements.

We seek in silico mechanisms that will provide plausible, mechanistic explanations of variability in BE. The requirements for models to be explanatory are well established [16,17]. We constructed an object-oriented, discrete event model that maps abstractly to two key components of a bioequivalence study: a dissolution test system and a study subject to whom originator product and test formulation were administered orally. We use small caps when describing subject features and components to avoid confusion and make clear that subjects cannot be the same as the BE study counterparts to which they map. Subject and its framework are illustrated in Figure 2. Because methods that follow are different from those used for conventional pharmacokinetic and pharmacodynamic modeling and simulation, some terminology is also new. Glossaries are available at (http://furm.org/glossary.html) and in [15].

### Iterative refinement protocol

**Figure 3:**
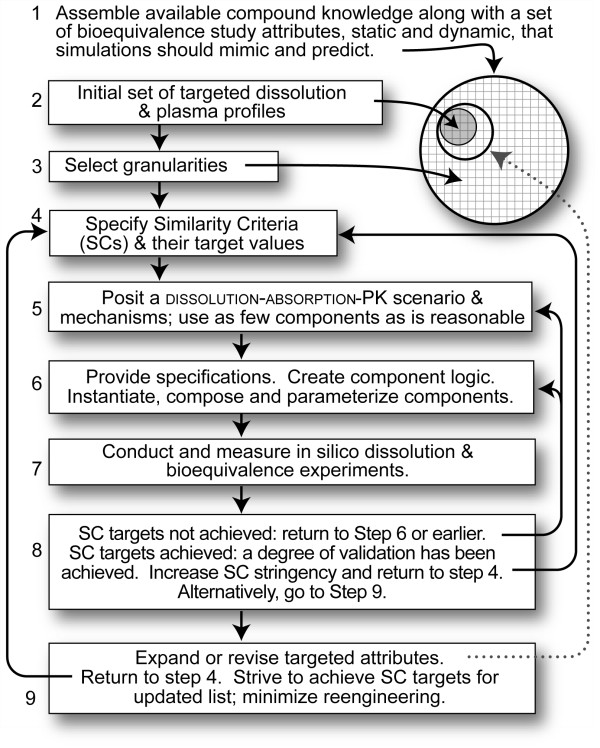
**Iterative Refinement Protocol was used to systematically develop and improve a subject and the outcomes of in silico bioequivalence studies.**

Simulation experiments must follow protocols, analogous to bioequivalence studies. We followed the IR Protocol in Figure 3, which is based on the scientific method: cycles of subject assembly; testing and evaluation; validation or falsification; assessment; cogitation; and feature or scenario revision. The process continues until prespecified SC are achieved or not. The protocol has features in common with protocols used for modeling and simulating complex ecological systems [18,19]. SC are discussed below. They typically begin weak and then are strengthened, as done in [20-22]. We used the IR Protocol successfully for different model types [20-23]. For this work, the attributes targeted include the product dissolution profile and features of the plasma drug level versus time profile (hereafter, simply plasma profile).

Mechanisms should be sufficiently complicated to achieve IR Protocol goals, but no more so. There is a strong impulse to add mechanistic details (specific regions of the intestine and flow through them, for example) before their need in achieving SC has been demonstrated, simply because we have knowledge of those details and evidence that they can contribute to plasma profile shape. Doing so too early is a mistake for two reasons. 1) As explained in [15], we are not trying to build an accurate, detailed model of typical subject. 2) It can lead to inscription error, which is the logical fallacy of assuming the conclusion and programming in (consciously or otherwise) aspects of the result we expect to see. Because a subject is an extensible, modular device, we know that we can add additional detail when needed, and doing so will be relatively straightforward. Adhering to that parsimony guideline encourages resisting making a subject any more complicated than needed to achieve current SC, while leaving unspecified the many other mechanisms that might influence the plasma profile. Adhering to that guideline is analogous to avoiding overparameteriztion of an equation-based model. We keep framework components simple by conflating fine-grained physiologic and anatomic details for which we have not yet demonstrated a need, and representing them collectively using abstract objects, spaces, and/or agents having relatively simple operating rules. Once SC and thus a degree of validation have been achieved, the behaviors of current components during simulation can be used for cross-model validation during development of alternative subjects having greater mechanistic detail, as done in [20,21,24].

When cycling through the IR Protocol, three attribute spaces are sampled and explored: subject mechanisms (types and properties of subject components, and their connection), parameter (including the mapping from time steps to clock time), and phenotype (a subject’s behavior space). For this project we focused on a narrow set of attributes, but as demonstrated in other projects [20-22,25], the focus can be a diverse set of phenotypic attributes. For complicated phenomena like a plasma profile during and following drug absorption, the reverse mapping from a phenomenon (e.g., curve shape) to plausible generators is one-to-many [15]; many, equally plausible mechanisms (networked events) can generate any one plasma profile. When dealing with people undergoing drug treatment, the mapping will be one-to-many no matter how much data we have, in part because of intra- and interindividual differences. Given ample resources, the ideal scientific strategy for gaining insight into mechanisms that may be responsible for a given plasma profile [15] would be to sample a variety of mechanisms and let them compete for survival through many IR Protocol cycles.

For specific mechanisms, like those depicted in Figure 2 and described below, only a subset of all possible parameter vectors (i.e., parameter value combinations) can achieve validation targets, in part because we are striving to be parsimonious, and some parameter value combinations are unrealistic or abiotic. However, when the number of attributes targeted is increased, that subset shrinks, sometimes to zero (see [20]); in the latter case, the mechanism is falsified. However, because of framework design, when that occurs, revision is easy. Similar shrinkage occurs when SC stringency is increased.

### System and SUBJECT components

We rely on object- and agent-oriented, discrete space, discrete event, software engineering methods [15,26,27] coupled with relational grounding (discussed below). The methods are analogous in several ways to established methods used for biological [28] and ecological research [18,19]. The basic methods have been described in [20,29,30]. Instructions for conducting in silico experiments and a description of the software are provided as Additional file 1. The following are provided in Additional file 2: a list objects, spaces, and their referents; descriptions of system components and an architecture diagram; and detailed descriptions of subject parameters.

A subject comprises a set of interconnected grid spaces and event mechanisms that map to different physiological features and processes (Figure 2). Our early, base model had three two-dimensional (2D), toroidal grid spaces, one each mapping to dissolution, GI, and plasma (plus all equilibrating tissues). All grid spaces are the same size: 100 x 100. Larger grid sizes did not measurably change subject outcome (results not shown). At each grid location is a simple container that can hold objects or numerical values. The spaces shaded differently within GI tract in Figure 2 illustrate that GI tract’s mechanistic properties can be made heterogeneous as needed by using multiple, independently parameterized grid spaces. The dissolution space is linked to both the GI tract and an Interaction Space. We hypothesize that the Interaction Space maps to individualized mechanistic heterogeneity that is a consequence of dosage form–GI tract interactions. GI tract and Interaction Space connect separately to plasma. The dissolution space and plasma are not connected. Not shown is somewhat less complicated subject, in which a “reservoir” space exists in place of Interaction Space. Drug can move from the dissolution space to GI tract and reservoir, and between GI tract and reservoir, but not from reservoir to plasma.

Framework components include a graphical user interface and data processor. The user interface allows the user to visualize and interactively probe subject and various parameter values during execution. The Data Processing Agent parses parameter files and experimental data for visualization and basic analysis. The agent is also responsible for making and recording measurements during simulation. Subject and the supporting framework are designed to be configurable, extensible, and modular so that additional, individualized components and detail can be added easily as needed.

### Simulating drug and its movement

Along one simulation path, drug can be represented as individual objects; along another path, it can be represented as numerical values. We used the numerical representation to simplify simulation and analysis. A numerical value designates the number of mobile drug objects at a grid location. For the reported studies, dose = 10,000. One drug maps to the amount of referent drug in a small aliquot of a referent fluid, plasma for example. Related works provide examples of using individual objects to represent compounds [20,25,30,31]. An advantage of using discrete objects is that each can carry identification information, such as a list of physicochemical properties of its referent, as done in [31]. The object representation enables one to obtain dynamic, fine-grained information on event histories and activities of individual drugs [25], however so doing significantly increases computation costs and complexity.

Time advances discretely in time steps (also called simulation cycles). One time step maps to several minutes; the exact number depends on other quantitative mappings, is subject-specific, and is specified by the parameter *XScale*. One time step maps to 0.5 h when *XScale* = 0.5. During each time step, all grid sites are updated. An update algorithm computes new values for all sites in sequential order: (0, 0), (0, 1), …(0, n), (1, 0), (1, 1), …(n, n). Once computing is complete, the update is finalized. Simulation results are recorded at the end of each time step. That data map to snapshots of referent system details taken at regular intervals. No assumptions are made about events occurring between time steps.

Inter-grid transfer and intra-grid relocation control drug movement. Inter-grid transfers occur between interconnected grids as illustrated in Figure 2. They are governed by a set of adjustable parameters. During each time step, a fraction of drug is transferred from one grid to another. In general, when Grid A has an incoming connection from Grid B and outgoing connection to Grid C, the amount of drug at site (*i*, *j*) is updated during time step *t* as follows:

(1) $$A_{i,j}\left(t+1\right)=A_{i,j}\left(t\right)+w_{\text{BA}}\left[f_{\text{BA}}B_{i,j}\left(t\right)\right]-w_{\text{AC}}\left[f_{\text{AC}}C_{i,j}\left(t\right)\right]$$

where *f*_BA_ specifies the fraction transferred from Grid B to A, *f*_AC_ is the fraction transferred from Grid A to C, *w*_BA_ and *w*_AC_ are Boolean variables with parameter-controlled probabilities. For example, drug transfer from GI tract to plasma is governed by the parameters *GtoPProb* and *GtoPFract*; they specify the probability of transfer occurring and the fraction of drug present at that site that is transferred during a time step. For each decision, a pseudorandom number is drawn from the uniform distribution; *w*_GP_ is set to ‘true’ if the drawn number < *GtoPProb*, otherwise ‘false’.

Intra-grid relocation simulates drug movement within (but not between) plasma, GI, and other structures. It uses a discrete approximation algorithm: $A_{i,j}\left(t+1\right)=\left[A_{i,j}\left(t\right)+d\left(N_{i,j}\left(t\right)-A_{i,j}\left(t\right)\right)\right]$, where *d* is the relocation rate, *t* is the relocation step counter, A_*i,j*_(*t*) is the drug amount at grid site (*i*, *j*), and N_*i,j*_(*t*) is the average drug amount across grid site (*i*, *j*) and its four neighboring sites. Higher relocation rates approximate well-stirred compartments (i.e., rapid distribution). Maximum relocation rate = 1 was used for all simulations. An iteration parameter sets the number of times the relocation algorithm executes per time step, and that was set to 2 for all simulations. Relocations execute independently of transfers.

System dynamics are a consequence of discrete events executed every time step. At the start of simulation, the dissolution grid is initialized with drug dose distributed across that space. Within a time step, grid-to-grid transfer events execute in the following sequence: 1) elimination from the plasma; 2) transfer from the GI tracts to plasma; and 3) transfer from dissolution to GI tract. All other events executed in pseudorandom order. Measurements are taken automatically every time step and recorded to output files at the end of simulation.

### Similarity criteria and quantitative comparisons

Similarity Criteria were specified arbitrarily, guided by examples of good and poor nonlinear mixed effect pharmacokinetic fits available in the literature. They are boolean tests that determine whether or not the simulated outcome is sufficiently similar to a feature (aspect) of the referent profile. Recent examples are provided in [25], which compared hepatic and simulated outflow profiles of diltiazem disposition in normal and diseased rat livers using similar, quantitative metrics. The SC specify that a simulated profile be within some factor of the referent values. They define upper and lower bounds around the target profile, and require that a specified number or ratio of simulated values occur within those bounds. In this study, we expected the consequences of a change in absorption details would be most evident in changes in ascending and descending portions of disposition profiles. Consequently, when specifying similarity, we gave more weight to those portions of the plasma profile. We specified two levels of stringency, starting with a medium stringency SC for baseline, initial subject development and testing. A higher stringency SC guided further refinement so that subject profiles matched referent more closely than did those obtained using conventional compartmental models (Additional file 2). The medium stringency SC used the following metrics for all nonzero values. For 0–10 h, all values must lie within a band ± 50% of referent values; in addition, four or more values must lie within ± 20% of referent values. For 10–48 h, all values must lie within ± 100% of referent values; in addition three or more values must lie within ± 30%. During iterative refinement, we also modified the mean in vitro dissolution profile, within limits, when doing so was needed to achieve plasma profile SC. The medium stringency SC for dissolution and referent profiles required that no more than two nonzero values lie outside ± 50% of referent values. Once we achieved the medium SC for all subjects, we applied the stringent SC, which tightened the threshold band for plasma profiles to require all nonzero values lie within ± 25%; in addition, four or more values must lie within ± 10% of referent values for the 0–10 h period.

A simple quantitative comparison metric was used to assess similarity between the simulated and subject profiles. We used the metric to guide selection of model parameterizations that provided for closer approximations to the referent profile. The metric gives an average of all values computed using the following formula:

(2) $$\exp\left(-\left|\left(y-y'\right)/y\right|\right)$$

where *y* is the referent value, and *y'* is the simulation value. Metric values closer to 1 indicate tighter approximations. The metric was applied to both the dissolution and plasma profiles.

We used dose fraction values for comparisons described above. Dose fraction refers to the fraction of initial, total drug objects at some location within the simulation. For example, with an initial dosage of 10,000 drug objects, a dose fraction of 0.005 translates to 50 drugs. We use dose fraction because it is unitless and enables direct superposition of in silico and clinical data. It also facilitates using relational than absolute grounding [32]. Comparisons were made on individual (vs. averaged) profiles; averaging over multiple runs did not provide statistically meaningful or useful insights.

### From simple to more complicated SUBJECTS

Adjustable delay parameters (initially *DtoGDelay* and *GtoPDelay*; later also *G2toPDelay*) were needed to better approximate observed plasma profile time lags and enable achieving the medium SC. However, when using only three grids, we failed to identify parameterizations to achieve the stringent SC. Major confounding factors included biphasic plasma profiles and the appearance of ratcheted or multiple peaks in plasma profiles. To achieve the stringent SC, we connected a second grid space to GI tract and called it reservoir; we refer to those models as r-subjects. We allowed drug to move between the reservoir and GI tract. So doing enabled r-subjects to produce sharper and multiple peaks. It also enabled achieving the stringent SC for some but not all subjects. Failure to achieve the stringent SC falsified that r-subject. While reviewing failed cases, we noted that plasma profiles fared poorly in matching multiple peaks in the 0–15 h period. We then created new subjects having two, independently parameterized grids (GI tract and Interaction Space in Figure 2) that together map to referent subject’s GI tract. So doing enabled simulations to better match peaks. Having dual grids enabled achieving the stringent SC for all eleven subjects. We refer to those models (Figure 2) as heterogeneous GI subjects, hereafter HGI subjects. Additional details and parameter descriptions are provided in Additional file 2.

In addition to the parameters already specified, subjects with GI tract and Interaction Space used the following parameters. *DtoGFract*, and *DtoGProb* define the dose fraction transferred and the probability of transfer from dissolution grid to both grids. *DiffGRatio* specifies the fraction transferred to GI tract and Interaction Space; for example, when *DiffGRatio* = 0.8, 80% of transferred drug goes to GI tract and 20% to Interaction Space. A difference in *DiffGRatio* (or another parameter) between the originator and test version of a subject instantiates an in silico counterpart of a subject-by-formulation interaction.

*GtoPFract*, and *GtoPProb* govern drug relocation from GI tract to plasma; *G2toPFract* and *G2toPProb* govern movement from Interaction Space to plasma. *PtoEDelay*, *PtoEFract*, and *PtoEProb* are the probabilities controlling drug elimination from plasma each time step. *YScale* is a scalar. It is applied to dose fraction in plasma to account for differences between dissolution and plasma concentrations measurements.

### Hardware and software

The framework code and instructions are available from the Corresponding Author. Subjects and supporting modules were implemented in Java using a multi-agent simulation library, MASON (http://cs.gmu.edu/~eclab/projects/mason). Batch simulation experiments were performed on a small-scale server. For model development, testing, and analysis, we used personal computers. We used R 2.7 (http://www.r-project.org) for data analysis and graph production.

## Results

Prior experience with this class of models provided informal heuristics for manually searching parameter space for parameter vectors that would enable achieving the medium SC. When cycling through the IR Protocol, we typically first adjusted parameters to mimic the dissolution profile. Next, we strove to mimic the plasma profile. As indicated by how SC are specified above, we placed more emphasis on matching C_max_ and T_max_ and less on matching the plasma profile tail, in part because C_max_ and T_max_ are emphasized in BE studies. For each originator-test pair, we completed simulations to first match the originator profile, and once successful, we shifted focus to matching the test profile in new, separate simulations. With simple subjects, we discovered parameterizations that enabled achieving the medium SC for all eleven subjects.

Having achieved the medium SC, we next focused on the stringent SC, which required greater similarity for the 0–10 h interval. For subjects 1 and 7, we located parameter vectors that enabled achieving the stringent SC for both the dissolution and plasma profiles of the test product. However, we failed to achieve the stringent SC for the remaining profiles, some of which exhibited noteworthy volatile patterns (e.g., subjects 5 and 8). Those failures falsified the simple subject mechanisms. We shifted to r-subjects. In so doing, we increased the model granularity, increased mechanistic detail, and expanded subject phenotype as reflected in plasma profiles. At that stage, because relevant individual subject physiological details were absent, we did not get into issues of mapping r-subject mechanisms to human reality: to do so would be purely speculative. The increased mechanistic detail enabled achieving the stringent SC for several additional cases (details not shown) but failed to do so for others. Again, those failures falsified the reservoir subject mechanisms.

**Figure 4:**
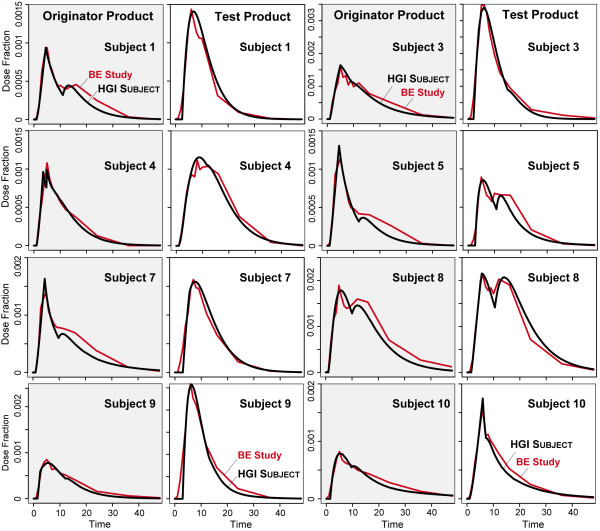
**Plasma profiles.** Profiles for three additional subjects are provided in Additional file 2. We used default parameter values to initialize HGI subjects and execute initial simulations. Grid size was set to 100 x 100, and probability parameters governing drug movement as specified in Table 1. Once initialized, the simulation was executed and stopped after a predefined number of time steps. Simulated plasma values were recorded each cycle and scaled in dose fraction to directly compare with the referent values. If the outcome failed to satisfy the prespecified SC, we adjusted parameter values and repeated simulation. We repeated the process for each referent profile until simulation measures achieved the SC. All simulations with Table 1 parameter values achieved the stringent SC. Red: referent plasma profile from the BE study; black: simulated profile from HGI subject.

We then shifted attention to HGI subjects. Other strategies for increasing event options within subjects could have been explored. Our task was simply to discover one that could achieve the stringent SC. We did not increase model granularity (relative to r-subjects), but we did marginally increase mechanistic detail, while also expanding subject phenotype. Again, we did not get into issues of mapping HGI subject mechanisms to specific GI details. The increased mechanistic detail enabled achieving the stringent SC for all cases. We located parameter vectors (Table 1) that produced the plasma profiles in Figure 4 that more closely resembled the observed profiles, and achieved the stringent SC. Having the additional Interaction Space feature was sufficient for approximating profiles with odd peaks like those observed in subjects 5, 8, and 10. Three additional matched profiles are provided in Additional file 2.

**Table 1:** **Parameter values for HGI subjects Order: originator/test**

		**Subject**							
**Parameter**^**1**^	**Default**	**1**	**3**	**4**	**5**	**7**	**8**	**9**	**10**
*XScale*	1	0.5	0.5/1.0	0.25/0.5	.125	.25/.5	.5	.125/.5	.125
*YScale*	120	330	220/120	120/330	220/110	110/330	110	110	110
*DtoGDelay*	1	1	1	1	1	1	1	1	1
*DtoGFract*	.1	0.1/0.2	.13/.33	.05/0.2	.03/.05	.05/.16	.13/.28	.05/.25	.025/.05
*DtoGProb*	.8	0.8	0.8	0.8	0.8	.8/.85	.8	.4/.9	.8
*DiffGRatio*	1	0.8/1	.65/.93	.94/.9	.75/.65	.6/1	.6/.4	.9	.88/.9
*GtoPDelay*	0	5/4	0	4/0	7/21	4/6	0/3	12/6	0/8
*GtoPFract*	.1	1.0/0.3	.78/.88	.065/.14	.25/.1	.34/.46	.65/.4	.2/.38	.12/.1
*GtoPProb*	.8	1.0/0.8	.78/.88	0.89	0.27/.3	.34/.46	.65/.4	.2/.38	.12/.1
*G2toPDelay*	20	32/20	18/15	19/23	96/86	39	20/21	97/26	0/58
*G2toPFract*	.1	0.3/0.1	0.2/0.5	1.0/0.3	0.12	.13	.23/.24	.11/.23	.05/.23
*G2toPProb*	.8	0.3/0.8	0.2/0.5	1.0/0.3	.13/.12	.13	.23/.24	.11/.23	.05/.23
*PtoEDelay*	0	7/0	10/3	14/7	36/0	18/11	1/11	20/0	35/48
*PtoEFract*	0.1	.25/.11	.14/.26	.69/.9	.125/.24	.15/.1	.14/.12	.22/.48	.11
*PtoEProb*	0.8	.6/.88	.8/.93	.3/.86	0.5/.4	1/.8	1/.9	.5/.4	.8

^1^ Additional parameter information is provided in Additional file 2.

## Discussion

A rationale for this new approach is that we can improve insight into the mechanisms responsible for differences in Figure 4 plasma profiles by making the individual mapping from simulated to actual profile concretizable. That can only be done if the simulated profiles are a consequence of actual, observable, processes. At the start of such a process (that is where we are with this report), the actual in silico processes needed for validation will necessarily be abstract and coarse grain.

A traditional, inductive, dissolution-absorption-pharmacokinetic model of the type used in nonlinear mixed effects analyses hypothesizes an explanation of patterns in plasma profile data. The mathematics describe data features predicted to arise from conceptualized mechanisms, which in turn are typically described in sketches and prose [1-3,6]. There is an unverifiable, conceptual mapping between equations and envisioned mechanisms [15]. The methods used herein are different. They provide three capabilities: 1) an independent, scientific means to challenge, explore, and better understand any inductive mechanism and, importantly, the assumptions on which it rests; 2) an additional experimental means of exploring, discovering, and testing the plausibility of subject-by-formulation interaction details at coarse grain level, along with causes of intra- and interindividual variability observed in bioequivalence study results; 3) a means to leverage the investment in BE studies and the research that preceded them by constructing and studying mechanistic analogues of dissolution and absorption processes contemporaneously with product development.

Measures of plasma during simulation experiments provide a test of the mechanistic hypotheses built into that subject. An acceptable similarity between in silico and BE study data is evidence that a concretizable mapping may exist between the dynamics occurring during simulation and corresponding dynamics thought to occur within that BE study subject, even though the actual events and processes in the two systems are different. To the extent that the mapping is accepted as realistic, we can posit that the implemented mechanisms may also have real counterparts.

However, given complex phenomena such as the profiles in Figure 4, there are, for a prespecified level of granularity, many, equally plausible biomimetic generators. To better understand intra- and interindividual variability, we will need to narrow the set of competing mechanistic explanations, and zero-in on causes of subject-by-formulation interactions, when present. To do that, we need modeling and simulation methods like those presented herein that are intuitive, heuristic, flexible, adaptable, and easily individualized [15,33]. Even though we present just one plausible, mechanistic explanation for each plasma profile, it is straightforward to develop others when that is needed. An understanding of these mechanisms may be useful in controlled-release formulation development to minimize the type of intra-subject variation observed in C_max_ for subjects 1, 2, 3, 5, 9, 10, and 11. So doing would improve in vivo absorption performance.

Subjects have been designed to use relational grounding [15,32] for maximum flexibility. For mappings to be quantitative, as in Figure 4, an additional model — a method of scaling; a quantitative mapping — is needed to relate a subject’s plasma profile directly to the referent plasma profile. That was done using the parameters *XScale* and *YScale*. For several subjects, achieving the stringent SC required varying the *XScale* and/or *YScale* values between the test and originator plasma profiles. *XScale* maps time steps to BE study time. By changing the *XScale* value, we alter the time granularity of simulation relative to in vivo time, which enables adjusting simulation for differences in the subject’s physiological condition (as influenced by stress, for example, or the previous day’s activities), which affect GI physiology, metabolism or other absorption and disposition related features. *XScale* does not influence product dissolution*.* A change in *YScale* maps to systemic variations in plasma concentration measurements between experiments, which may include changes in effective volume of distribution and bioavailability. It should be noted that changes in *XScale* and *YScale* values within or between subjects are evidence that the subject’s physiology changed between occasions. If we were to move these scaling models into each subject, we would immediately reduce subject flexibility, which is scientifically undesirable [32].

The levels of temporal, spatial, and mechanistic granularity (which control resolution) are somewhat arbitrary: they need to be sufficiently fine-grain so that a subject’s plasma profile meets the stringent SC. Granularity can be easily increased or decreased when that is needed. Because interactions within and between subject components are grounded relationally, an algorithm can be implemented when needed to automatically adjust parameter values to accommodate new levels of granularity so that the consequences of mechanisms and events can remain essentially unchanged.

One might object that the subject in Figure 2 is too abstract: distinguishable GI-like features are absent; there is no drug movement through sequential GI spaces, etc. Such features are absent because they were not needed to achieve the stringent SC. A scientific modeling and simulation good practice is to avoid inclusion of detail that is not part of the validation strategy. However, there is a scientific approach to drill down to additional, plausible, concrete, mechanistic detail. It requires expanding the list of targeted attributes and using the IR Protocol in the context of cross-model validation [24] to validate or falsify the need for that detail. Such attributes may include fine-grained details such as distinct cell types, enzymes, and transporters (see [25] for example). The approach is especially useful because of the scientific role played by experimentation on the current, validated subject analogue.

Further knowledge about specific formulation and dissolution details, which we do not have, can be used to specify additional SC that when met will shrink the space of plausible subject mechanisms, which may bring informative details into focus. That process may lead to identification of patient factors that correlate with subset membership. The insights are expected to enable developing an improved formulation. The subject model on which we focused represents the initial step in that direction. As relevant findings and data from in vivo dissolution become available, we may proceed to iteratively incorporate the information into subjects and achieve new validation. If successful, the descendant models could provide quantitative, mechanistic, clinically useful insight into how and why the in vitro dissolution differs (or not) from in vivo mechanisms. That insight could guide the design and development of formulations to optimize desired dissolution/absorption while minimizing adverse or otherwise undesirable characteristics.

## Conclusion

In summary, we used object-oriented, discrete event modeling and simulation methods to build and individually parameterize a subject — a software device — so that when events are measured during simulations, results mimic essential features of drug X dissolution profiles and individual plasma profiles measured during a BE study. In time, the proposed methods may be beneficial to drug development (e.g., controlled release) and to a reduction in the number of subjects needed per study plus faster regulatory review. For a new molecular entity, the strategy is expected to be useful during bridging studies (e.g., change in formulation from clinical to a new to-be-marketed version).

## Abbreviations

BE: Bioequivalence; C_max_: Plasma profile maximum; GI: Gastrointestinal; IR: Iterative refinement; SC: Similarity criteria.

## Competing interests

The authors declare that they have no competing interests.

## Authors’ contributions

SHJK and AJJ contributed equally. SHJK built the framework. SHJK, AJJ, and CAH contributed to refinement decisions. SHKJ, AJJ, and RH conducted simulation experiments. SHKJ, CAH, and AJJ wrote the manuscript. All authors read and approved the final manuscript. The views expressed are those of the authors and do not necessarily reflect the official views of FDA.

## Supplementary Material

Additional file 1 User instructions, and technical summary of software. Click here for file

Additional file 2 Additional subject profiles, and system details. Click here for file
