# Supplementary material for: Individualized, discrete event, simulations provide insight into inter- and intra-subject variability of extended-release, drug products
Source: Theor Biol Med Model. 2012 Aug 31;9:39. doi: 10.1186/1742-4682-9-39 (PMC3563477; doi:10.1186/1742-4682-9-39)
Supplement: Additional file 1 — User instructions, and technical summary of software. [file 1742-4682-9-39-S1.pdf]

SUPPLEMENTARY MATERIAL 2

Individualized, Discrete Event, Simulation Methods Provide Insight into Mechanisms of Inter- and Intra-Subject Variability of Reference and Generic, Extended-Release Drug Product

Sean H. J. Kim, Andre J. Jackson, Rim Hur, C. Anthony Hunt

The GridSim Software and Its Use

I. Installation

Before running the simulation, users need to download and install the Mason library. Because this library does not contain ParameterDatabase files, users need manually to download and add these files to the Mason library. Users should also download and install the JFree Chart and OpenCSF libraries.

II. Getting Started

When the user runs the “GridSimMain.java” file, four pop-up GUI windows, by default, will appear as shown in Figure 1: the Dosage Chart, GridSimM, Plasma Chart, and Simulation Info.

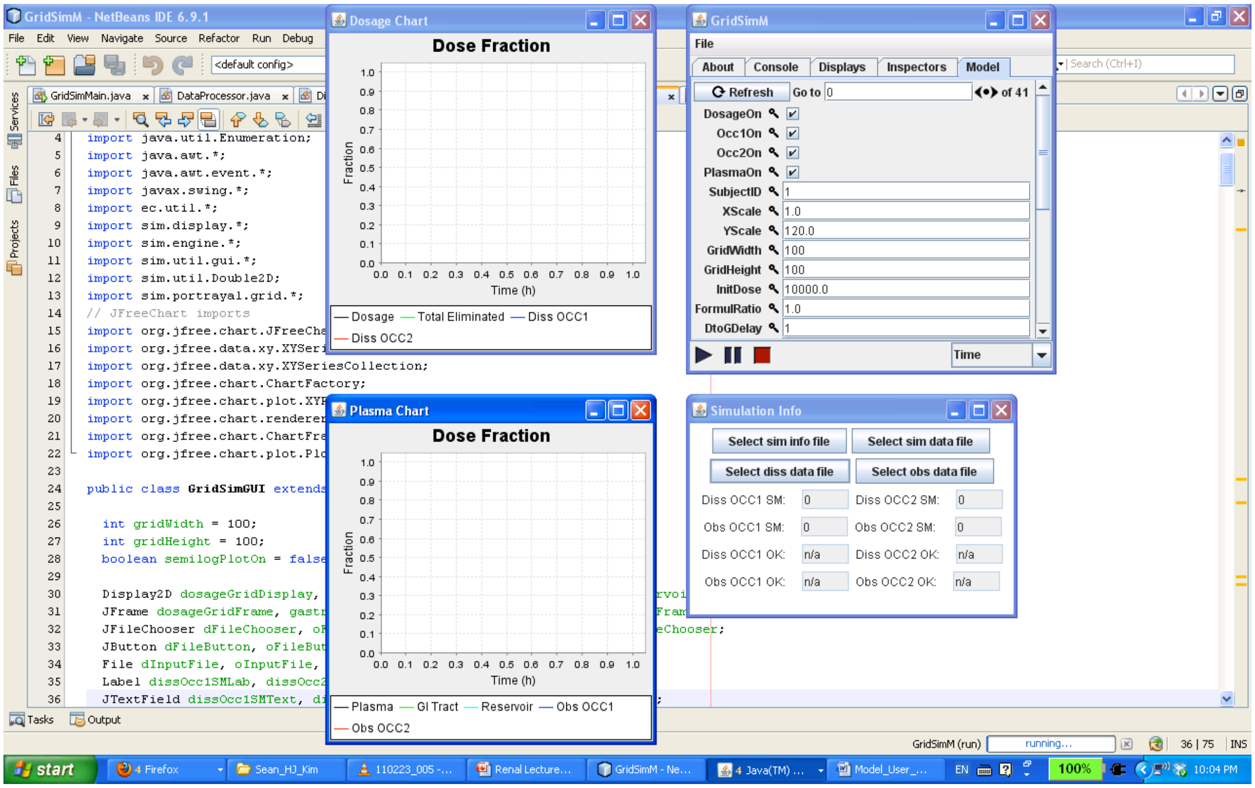

Figure 1: Dosage Chart, GridSimM, PlasmaChart, and Simulation Info GUI windows by default.

III. GUI Windows and Panels

Of those four GUI windows, the Simulation Info window allows users to read and upload data files. Figure 2 is a magnified screenshot of the Simulation Info GUI Window. To upload dissolution data, users can click on the "Select Diss Data File." Similarly, by clicking on the "Select Obs Data File," users can upload plasma concentration data. Users can also upload data files to which the program can write. The "Select Sim Info File" button allows users to record and write the values of the following parameters: "DtoG_D; DtoG_F; DtoG_P; GtP_D; GtP_D; GtoP_F; GtoP_P; PtoE_D; PtoE_F; PtoE_P; SM_DS_1; SM_DS_2; SM_OB_1; and SM_OB_2; Cycle. In a similar fashion, by clicking on the "Select Sim Data File" button, users can upload data files to which the program can record and write the values of the following parameters and concentration levels: Cycle; Dosage; GI; Plasma; and Elimination. Once the simulation is completed, users can check the "Diss OCC1 OK," "Diss OCC2 OK," "Obs OCC1 OK," and "Obs OCC2 OK" parameters to see whether the simulated values met the stringency criteria. The parameter value of "YES" indicates that the corresponding set of simulated values is within a given tolerance range.

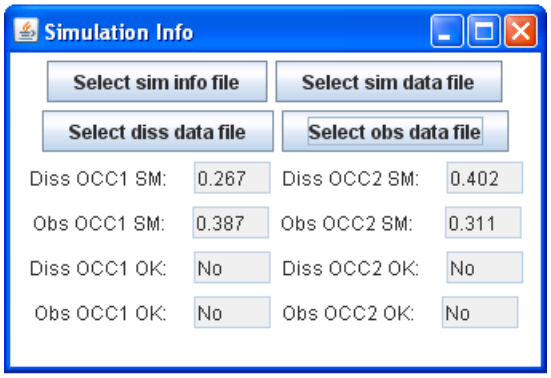

Figure 2: Simulation Info GUI Window

There are five different types of compartments within each space the drug of interest can exist: Dosage Space; Gastro Intestinal Space; Reservoir Space; Plasma Space; and Elimination Space. By manipulating the parameter values of the GridSimM GUI Window, users can simulate the movements of the drug molecules among different compartments. In the *Model* panel of the GridSimM GUI Window, the user should first enter the subject identification number. The *Model* panel is shown in Figure 3. To enable the Dosage Chart GUI window and the Plasma GUI Window, respectively, users need to check the "DosageOn" and "PlasmaON" checkboxes. To simulate the first data curve and the second data curve, users need to check the "OCC1ON" and "OCC2ON" checkboxes respectively.

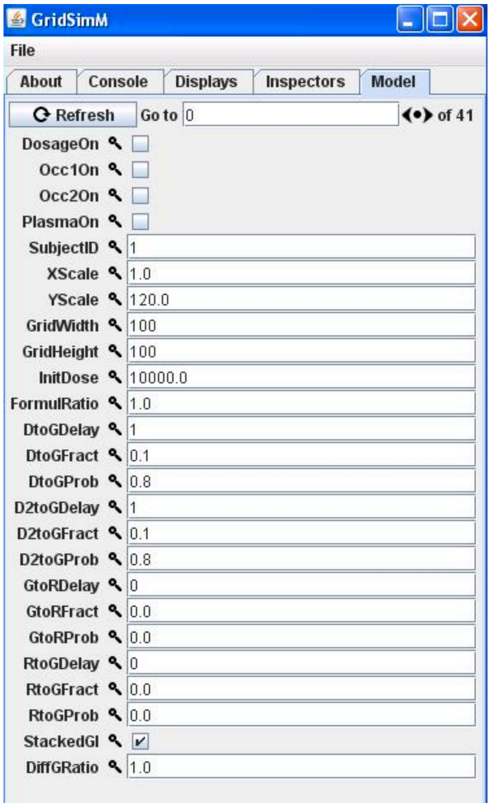

Figure 3: the 1st page of the *Model* panel

Three important variables can be manipulated to control the transfer processes of the drug molecules from one compartment to another: a time delay; a fraction value; and a probability value. After the amount of time given by the time-delay variable has passed, and the computer-generated random value during the given cycle is smaller than the user-given probability value, the program transfers per cycle the user-given fraction value of the drug molecules from the source compartment to the target compartment. All the time delay values, fraction values, and probability values are controlled from the *Model* panel.

When the “StackedGI” parameter is turned on in the *Model* Panel, users can add the second Dosage and the second Gastro Intestinal spaces to the simulation. These additional compartments represent heterogeneous spaces in the dissolution process as well as in the gastrointestinal absorption process. The parameter “DiffGRatio” determines the weight of influence of each heterogeneous space. For example, when the value of “DiffGRatio” is 0.9, a weight of 90% is conferred to the Dosage 1 space, and a weight of the remaining 10% to the Dosage 2 space.

The *Model* panel has two pages. By clicking on the arrows on the upper-right corner, users can navigate between the two pages. Figure 4 shows the second page of the *Model* panel.

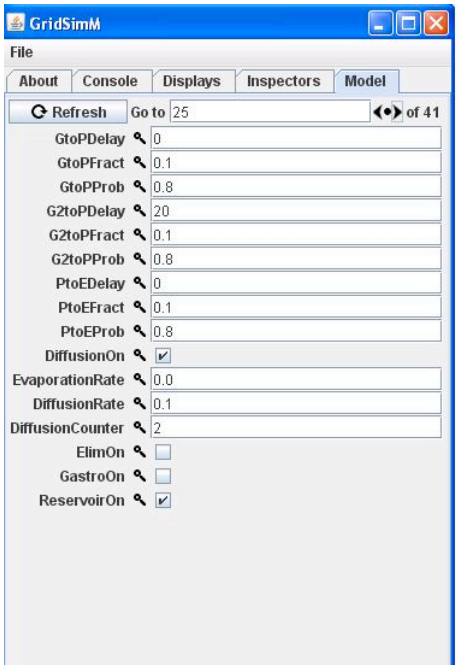

Figure 4: the 2nd page of the *Model* panel

Additionally, users are able to change the effects of diffusion by checking the "Diffusion On" button, then changing the values of "Evaporation Rate," "Diffusion Rate," and "Diffusion Counter."

The *Console* panel, as shown in Figure 5, allows users automatically to stop the simulation at a user-given step or stop time. This panel also allows users to manipulate other values such as the Thread Priority.

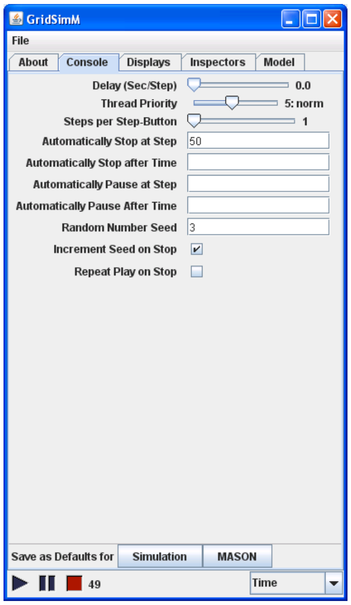

Figure 5: the *Console* panel

The *Display* panel, as shown in Figure 6, allows users to show or hide GUI windows. Figure 6 shows that in addition to the GridSimGUI window itself, the three bold-lettered GUI windows–i.e., Simulation Info, Dosage Chart, and Plasma Chart–are open. Users can show or hide the GUI Windows listed on the *Display* panel by clicking on the "Show" or "Hide" buttons. The "Dosage," "GI Tract," "Reservoir," and "Plasma" GUI windows are partly accessory windows that visually show the changes in drug concentrations in the corresponding compartments.

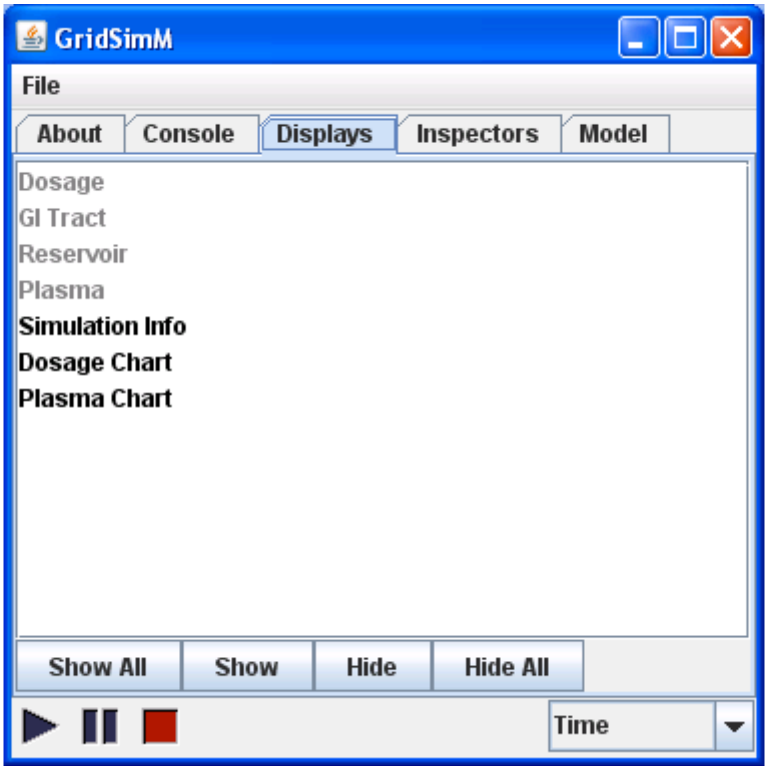

Figure 6: The *Display* panel

The Dosage Chart GUI Window, as displayed in Figure 7, shows the fraction amount of the drug molecules remaining in the Dosage space as well as the fraction of drug molecules eliminated. The black line represents the simulated curve; the blue line represents the first set of data; the red line represents the second set of data; and the green line represents the fraction of drug molecules eliminated (as shown in the legend.)

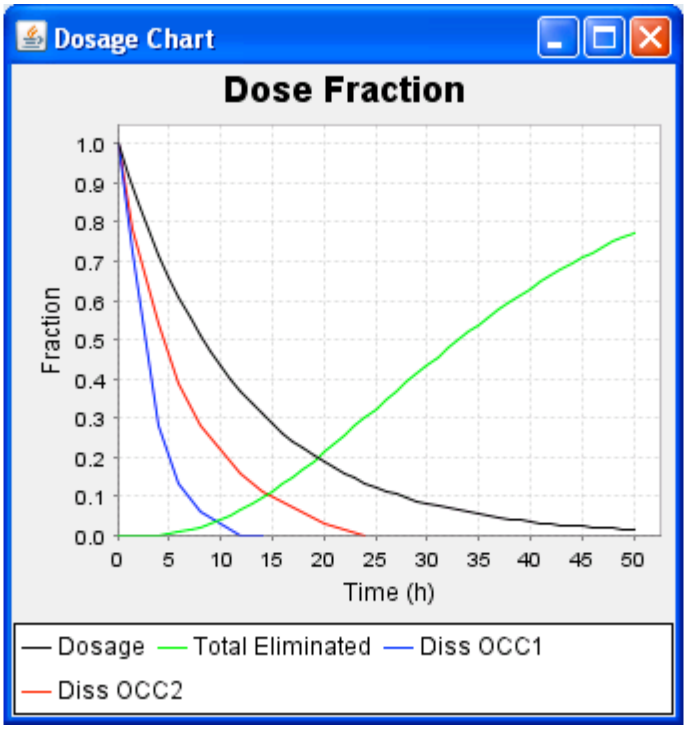

Figure 7: the Dosage Chart Window

Similar to the Dosage chart GUI Window, the Plasma Chart Window, as displayed in Figure 8, shows the simulation as well as the data curves of the drug molecules in the Plasma compartment.

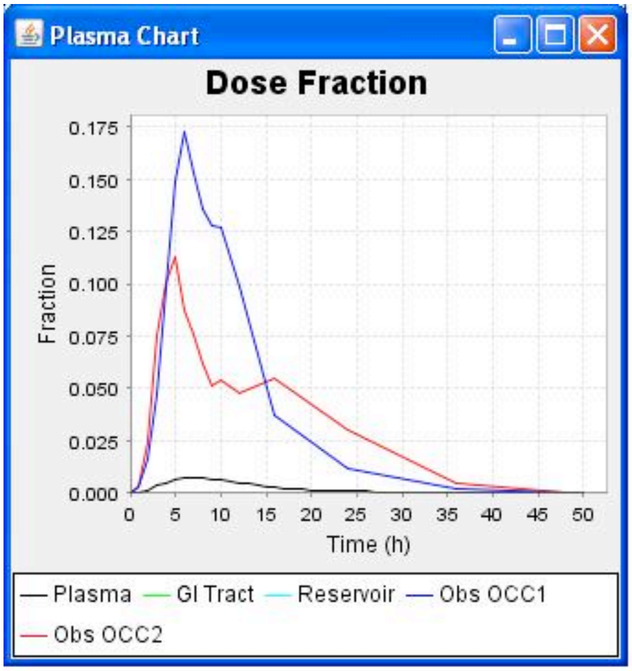

Figure 8: Plasma Chart GUI Window

IV. Important Methods

One of the important methods of the GridSim class is *transferGridVals (DoubleGrid2D source, DoubleGrid2D target, double fract, double prob)*. This method transfers drug molecules from the source space to the target space based on user-provided fraction and probability values. The *withdrawGridVals(DoubleGrid2D source, double fract, double prob)* method, on the other hand, simulates the elimination of drug molecules from a source space. Additionally, the *checkSimpleSM(int categ, int occ)* method checks the simulated values and informs users whether those values have met the stringency criteria.
