# Supplementary material for: Individualized, discrete event, simulations provide insight into inter- and intra-subject variability of extended-release, drug products
Source: Theor Biol Med Model. 2012 Aug 31;9:39. doi: 10.1186/1742-4682-9-39 (PMC3563477; doi:10.1186/1742-4682-9-39)
Supplement: Additional file 2 — Additional subject profiles, and system details. [file 1742-4682-9-39-S2.pdf]

ADDITIONAL FILE 1

Individualized, Discrete Event, Simulations Provide Insight into Intra- and Interindividual Variability of Extended-Release, Drug Products

Sean H. J. Kim, Andre J. Jackson, Rim Hur, C. Anthony Hunt

I. Preliminaries

1. Product details

The drug is BCS class I (highly soluble and highly permeable), and it is almost completely absorbed after oral administration. However, it undergoes high first pass metabolism by the liver, and on average, only about 25% reaches the systemic circulation. The kinetics are linear. Both the referent and test formulations are capsules filled with fast and slow dissolving beads.

2. Noncompartmental analysis

The data obtained from the bioequivalence study was analyzed using noncompartmental methods to assure that the results were similar to those obtained using the stringent SC. Analysis was done using Proc GLM in SAS. The 90% CI of 126-156 (ng/mL) for the test product C_{max} clearly implicates that it has much faster absorption than the referent formulation.

II. In Silico Modeling

1. GridSim and SUBJECT details

We developed a new, in silico modeling framework, which uses an open source, multi-agent simulation software library MASON (Luke et al., 2005). Here and in Additional File 2, we refer to the Framework as GridSim to distinguish it from other frameworks used by other projects. GridSim is object and agent oriented (Hunt et al., 2009), and supports discrete event, discrete time simulation (Zeigler et al., 2000). The MASON library provides a general purpose, discrete event simulation engine that includes a simulation clock, events queue handler, and random number generators (RNGs). GridSim uses that engine to execute simulation as detailed below.

As a pharmacological modeling framework, GridSim has basic components and features for in silico experimentation and analysis, including a data processor and graphical user interface (GUI). The data processor is a software agent that handles both input and output data. The agent parses referent data into internal data structures for visualization and analysis. It uses opencsv (http://opencsv.sourceforge.net) to translate CSV data into time series and store in vector data structures. Data access is allowed only via accessor functions (e.g., *getRefDataFractEnum*, *getRefDtaValFractAt*). GridSim GUI is a visualization wrapper that extends MASON's GUIState class. It uses GUIState's interactive console to handle user interaction, and Java's awt package to create graphics for charting and file selection. Line charts are drawn during simulation using an open source, Java charting library JFreeChart (http://www.jfree.org/jfreechart).

At the core of framework is a pharmacologically responsive, virtual patient model we call SUBJECT. The current version consists of a set of interconnected 2D square grids and event mechanisms that map to different physiological features and processes. The grids are object variables of MASON's DoubleGrid2D type. Variable values can be retrieved or updated using accessor and mutator functions. The model implements a step function scheduled at every simulation time step, which comprises a set of logic (procedural) statements that effect inter-grid

DRUG transfer and grid updates. A separate function (method) defines intra-grid movement, which executes a simple, discrete approximation algorithm for diffusion. The function is automatically scheduled to execute a prespecified number of times at each simulation time step. For both step functions, buffer grids are used to temporarily store new grid values (t+1) computed from current values at time step t. The model extends MASON's SimState and Steppable classes that automatically handle event scheduling and time step advances.

2. In silico components

The following are in silico objects with referent definitions, which are denoted in small caps to distinguish from their real-world counterparts.

- COMPOUND: a chemical compound
- DISSOLUTION: primary sites where drug dissolution occurs
- DOSE: a specific quantity of drug administered
- DRUG: a chemical substance used for therapeutic purposes (herein, we use the terms DRUG and COMPOUND interchangeably)
- GI TRACT: gastrointestinal (GI) tract
- PLASMA: blood plasma
- RESERVOIR: non-metabolizing sites where drug is sequestered from absorption
- SUBJECT: human subject in bioequivalence study

3. Model parameters

The following are model parameters that are accessible to the user. Default values shown can be modified before or during simulation. Changes in the following parameters take effect in a new simulation run: *GridWidth*, *GridHeight*, *InitDosage*, *MaxCencentration*, *DiffusionOn*, and *DiffusCounter*. Changes in all other parameters take effect immediately in the current simulation.

- *GridWidth, GridHeight*: grid space width (x-axis) and height (y-axis) applied to all grid spaces; default value = 100. Coordinates are indexed from 0 (e.g., $0 \leq x < GridWidth$).
- *XScale*: scalar factor used to map simulation cycles to real time (h); default value = 1. For example, one simulation cycle maps to 2 h when *XScale* = 2.
- *YScale*: scalar factor applied to the dose fraction in plasma to account for differences between dissolution and plasma concentration measurements; default value = 120.
- *InitDosage*: initial concentration value of the dissolution grid sites; default value = 0.02.
- *MaxConcentration*: the value at which the concentration color for visualization is maximally intense. Concentration values above the ceiling are painted at the same, maximal intensity. Usually the parameter is set to *InitDosage* value; default value = 0.02.
- *DtoGDelay*: initial delay (number of simulation cycles) before initiating simulated drug transfer from the dissolution grid to GI TRACT; default value = 1.
- *DtoGFract*: the fraction of simulated drug concentration transferred from *DissolutionGrid*[*i*][*j*] to *GITractGrid*[*i*][*j*], where $i < GridWidth$ and $j < GridHeight$. The amount transferred is *DosageGrid*[*i*][*j*] x *DtoGFract*. Valid range is between 0 and 1 inclusively; default value = 0.1.
- *DtoGProb*: the probability of transfer from *DissolutionGrid*[*i*][*j*] to *GITractGrid*[*i*][*j*], where $i < GridWidth$ and $j < GridHeight$. At the start of a transfer event, a pseudo-random number, $0 \leq p \leq 1$, is generated. Transfer occurs if $p \leq DtoGProb$; otherwise no transfer occurs from the site (i, j) in that simulation cycle. Valid range is between 0 and 1 inclusively; default value = 0.8.

- *DiffGRatio*: percentage of simulated drug fraction transferred to the primary GI TRACT grid; default value = 1. For example, when *DiffGRatio* = 0.8, 80% of the simulated drug fraction is transferred to the primary GI TRACT, and 20% to the secondary grid.
- *GtoPDelay, GtoPFract, GtoPProb*: the initial delay, fraction transferred, and the probability of transfer from the GI TRACT to PLASMA. Default values are 0, 0.1, and 0.8, respectively.
- *G2toPDelay, G2toPFract, G2toPProb:* the initial delay, fraction transferred, and the probability of transfer from the secondary GI grid to PLASMA. Default values are 20, 0.1, and 0.8, respectively.
- *GtoRDelay, GtoRFract, GtoRProb:* the initial delay, fraction transferred, and the probability of transfer from the GI TRACT to reservoir space. Default values are 0 (no reservoir).
- *RtoGDelay, RtoGFract, RtoGProb:* the initial delay, fraction transferred, and the probability of transfer from the reservoir space to GI TRACT. Default values are 0 (no reservoir).
- *PtoEDelay, PtoEFract, PtoEProb*: the initial delay, fraction eliminated, and the probability of elimination from the PLASMA grid. Default values are 0, 0.1, and 0.8, respectively.
- *DiffusionOn*: Boolean value indicating whether simulated diffusion occurs within each grid space; default value = true.
- *EvapRate*: the loss rate, which specifies the fractional concentration amount evaporated (i.e., dissipated) during simulated diffusion. Valid range is between 0 and 1 inclusively; default value = 0 (no evaporation).
- *DiffRate*: the diffusion rate, which abstractly corresponds to diffusivity or diffusion coefficient. Valid range is between 0 and 1 inclusively; default value = 0.1.
- *DiffusCount*: the number of diffusion step iterations executed per simulation cycle. Parameter value can be any positive integer; default value = 2.
- *DissOn, GITractOn, PlasmaOn, ElimOn:* Boolean values indicating whether the respective grid contents are tracked and graphed visually. Default value = true.

4. Program execution

SUBJECT is an executable, object-oriented model written in Java. It is not a formal mathematical model. Differences between the two model types are explained in (Fisher and Henzinger, 2007; Hunt et al., 2008). Here we focus on program execution details.

When the program starts execution, an interactive console is loaded automatically to allow user to select input files and/or specify parameter values. At the start of a simulation, a SUBJECT is instantiated and parameterized according to the user specifications. All grid sites are initialized to zero, except the DISSOLUTION sites that are initialized to *InitDosage*. Referent data are parsed by the data processor and stored as time series vectors to allow access for charting during simulation. The step functions for inter- and intra-grid DRUG movements are added to the event schedule (by calling SimState's *scheduleRepeating* function) to be executed at every time step. A new RNG instance (MASON's MersenneTwisterFast) is created with a seed set to system time. Once the initialization completes, the simulation executes for a prespecified number of cycles or until the user selects to halt execution. At every cycle, the two (intra- and inter-grid) step functions execute in a pseudorandom order to update grid site values, and in silico measurements are made and stored in temporary data files. We emphasize that model execution is not about determining or converging to a mathematical solution. A simulation simply repeats execution of the algorithms (sequences of logic instructions) that specify inter- and intra-grid DRUG transfers,

and update grid sites with computed values. For example, the following pseudocode computes updated values of the PLASMA grid sites:

```
for (int i = 0; i < PlasmaGrid.width; i++) {
   for (int j = 0; j < PlasmaGrid.height; j++) {
      If (MersenneTwisterFast.nextDouble() < GtoPProb) {
         PlasmaGrid.field[i][j] += GITractGrid.field[i][j] * GtoPFract;
         GITractGrid.field[i][j] -= GITractGrid.field[i][j] * GtoPFract;
      }
   }
}
```

Similar sequences update other grid spaces. The current model implements no parallelization: the inter-transfer algorithm executes to completion before intra-transfer starts, and vice versa. PLASMA DOSE fraction values plotted are the total amount of DRUG in the PLASMA grid at sequential time points. The in silico measurements are written to persistent data files when the simulation ends. A new SUBJECT is instantiated for each simulation run.

References

[1] Fisher J, Henzinger TA (2007) Executable cell biology. Nat Biotech 25: 1239-1249.
[2] Hunt CA, Ropella GE, Lam TN, Tang J, Kim SH, Engelberg JA, Sheikh-Bahaei S (2009) At the biological modelling and simulation frontier. Pharm Res 26(11): 2369-2400.
[3] Hunt CA, Ropella GE, Park S, Engelberg J (2008) Dichotomies between computational and mathematical models. Nat Biotech 26: 737-738.
[4] Luke S, Cioffi-Revilla C, Panait L, Sullivan K, Balan G (2005) MASON: a multi-agent simulation environment. SIMULATION 82(7): 517-527.
[5] Zeigler BP, Kim TG, Praehofer H (2000) Theory of modeling and simulation: integrating discrete event and continuous dynamic systems. San Diego: Academic Press.

Supplemental Figures

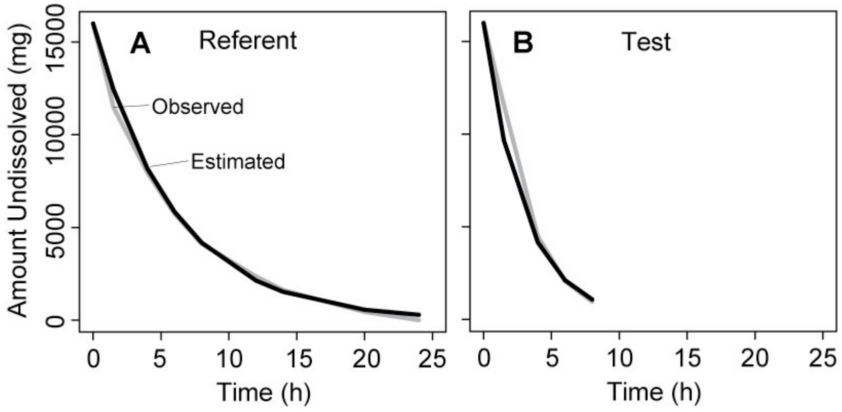

Figure S1. Dissolution curves. **(A)** Mean dissolution of the originator product. **(B)** Mean dissolution of the test formulation, which dissolved almost completely within 8 h. Observed: gray; estimated (used in simulations): black.

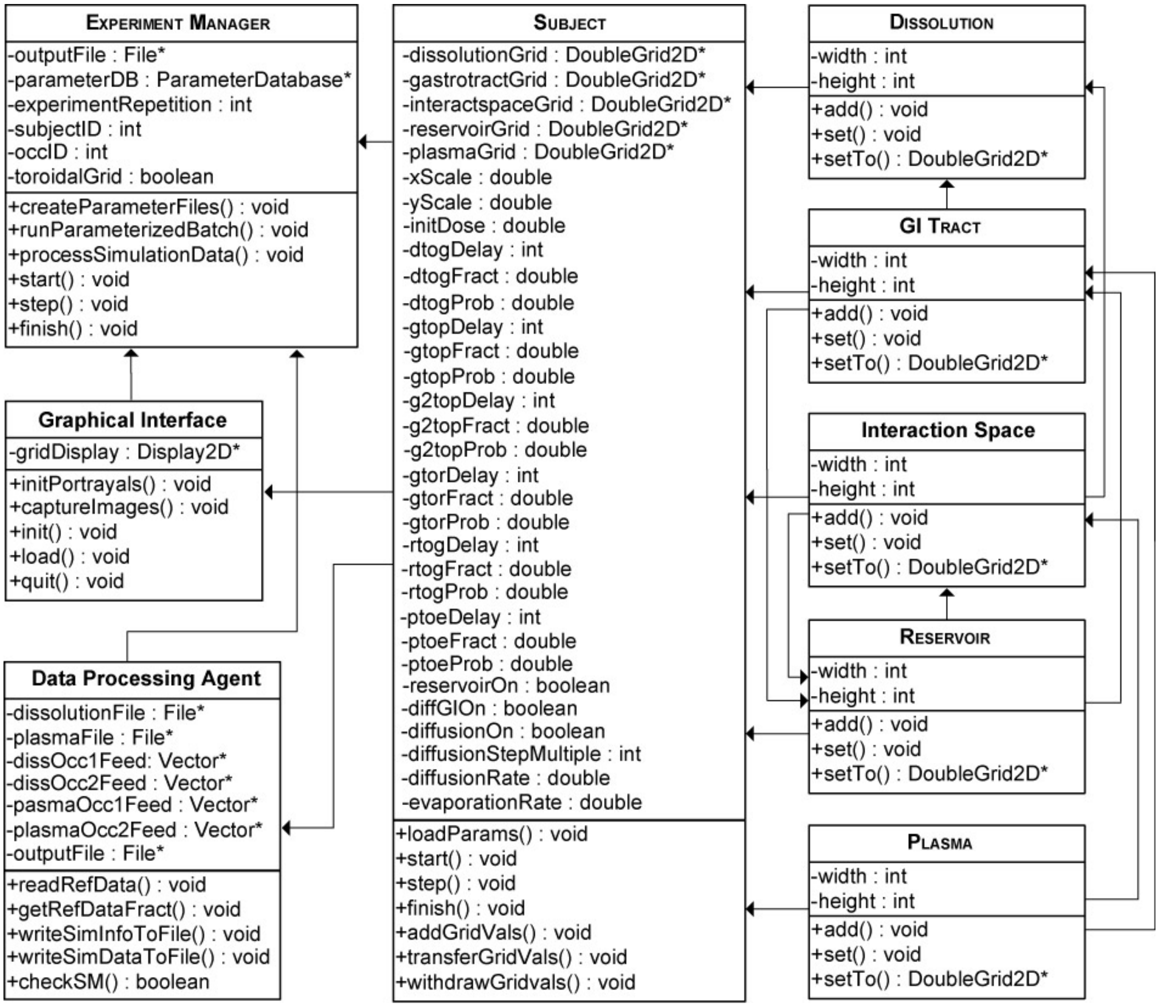

Figure S2. System components and architecture. Arrows indicate component dependencies and interactions. We use small caps to distinguish in silico components from their real-world counterparts. EXPERIMENT MANAGER is a top-level software agent that manages and executes simulation experiments, which simulates a scientist conducting experiments. The agent directly controls the graphical interface, data processing agent, and SUBJECT. Data processing agent parses referent data for visualization and analysis, and records measurements during simulation. It corresponds to laboratory instruments for observation and measurement, and has direct access to SUBJECT during simulation. SUBJECT is a virtual patient model, which consists of a set of interconnected 2D grids and event functions that abstractly map to physiological features and processes. Within SUBJECT, individual grids are connected as indicated by arrows. DISSOLUTION and GI TRACT represent primary sites where simulated dissolution and absorption occur. Interaction space is an optional compartment, which emulates non-homogeneous, locally differential absorption. Another option is RESERVOIR, which sequesters DRUG from GI TRACT; no ABSORPTION occurs within RESERVOIR. After ABSORPTION and distribution into PLASMA, DRUG is eliminated at a rate governed by the parameters *PtoEFract* and *PtoEProb*. *: predefined Java or MASON library object types; -: object variable or parameters; +: object functions.

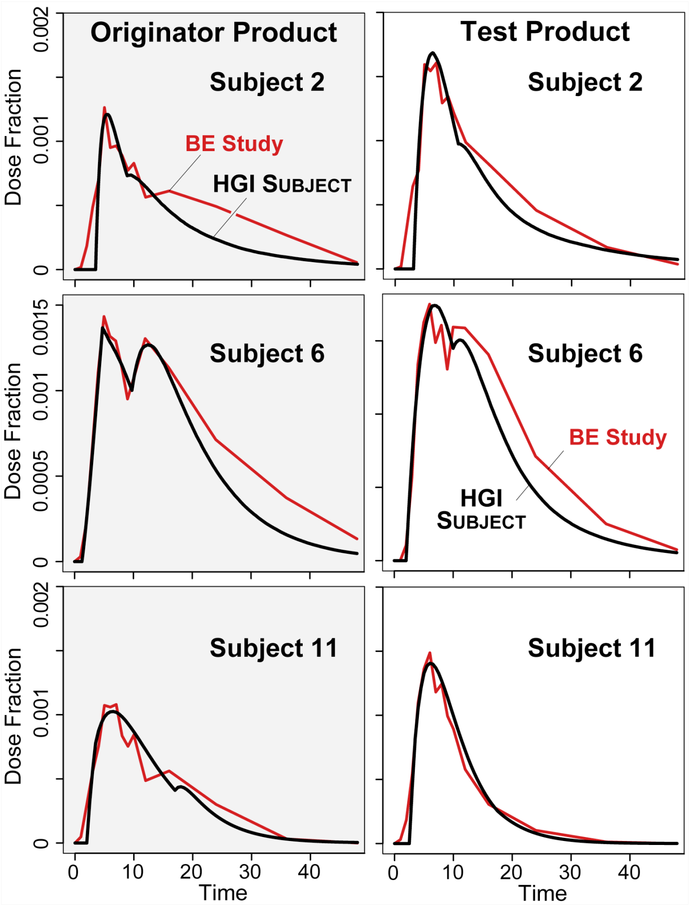

Figure S3. Additional plasma profiles. HGI SUBJECTS used Table S2 parameter values. All simulations achieved the stringent Similarity Criteria.

Supplemental Tables

Table S1. Experimental maximum plasma concentration (C_{max}), area-under-the-curve to the last measured time point *t* (AUC_{0-t}), and area-under-the-curve to time infinity (AUC_{inf}).

Parameter	Referent	Test	90% CI
C_{max} (ng/ml)	1635	2295	126-156
AUC_{0-t} (ng/ml*h)	2771	3334	111-131
AUC_{inf} (ng/ml*h)	2886	3388	108-127

Table S2. GridSim parameter values used to achieve stringent similarity criteria for Subjects 2, 6, and 11 profiles shown in Fig. S3. The same value was used to fit the referent and test profiles unless denoted (originator/generic).

		**Subject**		
**Parameter**	**Default**	**2**	**6**	**11**
GridWidth	100	100	100	100
GridHeight	100	100	100	100
XScale	1	0.125	0.125	0.5
YScale	120	120	110	330
DtoGDelay	1	1	1	1
DtoGFract	0.1	0.1/0.3	0.026/0.05	0.1/0.2
DtoGProb	0.8	0.2	0.8	0.8
DiffGRatio	1	0.6	0.55/0.65	0.94/0.98
GtoPDelay	0	28	10/16	4/5
GtoPFract	0.1	0.3/0.21	0.2/0.18	0.72/0.54
GtoPProb	0.8	0.33/0.2	0.2	0.72/0.54
G2toPDelay	20	71/86	78/80	34/35
G2toPFract	0.1	0.09	0.11/0.1	0.55/0.3
G2toPProb	0.8	0.09/0.08	0.11/0.1	0.55/0.3
PtoEDelay	0	0	38/0	7
PtoEFract	0.1	0.15/0.1	0.045/0.08	0.13/0.14
PtoEProb	0.8	0.45	1/0.47	0.8/0.78
EvapRate	0	0	0	0
DiffRate	0.1	0.1	0.1	0.1
DiffusCount	2	2	2	2
